# Functional imaging in lung cancer

**DOI:** 10.1111/cpf.12104

**Published:** 2013-12-01

**Authors:** S W Harders, S Balyasnikowa, B M Fischer

**Affiliations:** 1 Deparment of Radiology, Aarhus University HospitalAarhus, Denmark; 2 Diagnostic Radiology Department, N.N. Blokhin Russian Cancer Research Center RAMSMoscow, Russia; 3 Department of Clinical Physiology, Nuclear Medicine and PET, RigshospitaletCopenhagen, Denmark

**Keywords:** diagnosis, diffusion magnetic resonance imaging/methods, lung neoplasms/diagnosis, neoplasm staging, positron emission tomography and computed tomography/methods, tomography, x-ray computed/methods

## Abstract

Lung cancer represents an increasingly frequent cancer diagnosis worldwide. An increasing awareness on smoking cessation as an important mean to reduce lung cancer incidence and mortality, an increasing number of therapy options and a steady focus on early diagnosis and adequate staging have resulted in a modestly improved survival. For early diagnosis and precise staging, imaging, especially positron emission tomography combined with CT (PET/CT), plays an important role. Other functional imaging modalities such as dynamic contrast-enhanced CT (DCE-CT) and diffusion-weighted MR imaging (DW-MRI) have demonstrated promising results within this field. The purpose of this review is to provide the reader with a brief and balanced introduction to these three functional imaging modalities and their current or potential application in the care of patients with lung cancer.

## Introduction

Lung cancer, of which non-small-cell lung cancer accounts for 80–90% (D'addario *et al*., 2010; Pastorino, 2010), represents the leading cause of cancer mortality worldwide, each year causing the death of approximately 1·2 million people. The incidence of lung cancer is closely related to smoking patterns, and thus, a dramatically increasing incidence and mortality have been observed in China (Yang *et al*., 2004), while the incidence in Western countries has been expected to decrease, but keeps slowly increasing – mainly due to the increased incidence among females and the elderly (Palshof & Jakobsen, 2010).

During the last three decades, the approach towards lung cancer has changed from defeatistic to slightly optimistic, with focus on early diagnosis and precise staging, as the fundament for a growing number of therapy options. These advances have, however, resulted in only slightly improved survival; for example, in Denmark, overall survival for patients with lung cancer has improved with a total of 6 months during the last 10 years (Palshof & Jakobsen, 2010; Coleman *et al*., 2011). This improvement is mainly attributed to the increased number of therapy option and improved surgical techniques, but also faster diagnosis and improved staging are thought to play a role (Palshof & Jakobsen, 2010).

The purpose of this report is to review and discuss current literature on functional imaging in lung cancer, by functional imaging meaning modalities that provide us with more information than simple anatomy. We will try to provide the reader with a balanced introduction to one well-known and two newer functional imaging modalities in lung cancer (Table1), namely integrated positron emission tomography and CT with ^18^F-FDG (FDG-PET/CT) assessing tissue metabolism, dynamic contrast-enhanced CT (DCE-CT) estimating tumour blood flow and blood volume and diffusion-weighted magnetic resonance imaging (DW-MRI) exploring cellular density (cellularity and the integrity of cell membranes.

**Table 1:** Summary of technical details and comparison of PET/CT, DCE-CT and DW-MRI.

Modality	FDG-PET/CT	DCE-CT	DW-MRI
Principle	Describing tumour metabolism using the radioactive tracer FDG	Visualizes and measures tumour blood flow and blood volume.	Shows diffusion of water molecules within the tissue conditioned by cellular density
Patient preparation	4- to 6-h fast 1-h rest after injection	30-min rest after injection	No preparation
Examination time	20 min	2 min	15 min (per bed position)
Postprocessing	10 min	10-15 min	10 min
Quantification	Possible (SUV), but not necessary	Possible, but unreliable	Possible (ADC), but not very useful
Radiation dose	5–15 mSv, dependant on CT protocol and FDG dose	10–20 mSv, dependant on protocol	No radiation
Strength	High throughput Well validated Very sensitive	Functional modality High accuracy	No radiation No preparation No contrast Sensitive
Limitations	FDG not specific for cancer Radiation dose	Respiratory motion Reproducibility Radiation dose	Low SNR Respiratory motion and cardiac pulsation cause artefacts

DCE-CT, dynamic contrast-enhanced CT; DW-MRI, diffusion-weighted magnetic resonance imaging; FDG-PET/CT, integrated positron emission tomography and CT with ^18^F-FDG; ADC, apparent diffusion coefficient; SUV, standardized uptake value.

## Techniques and limitations

### Integrated positron emission tomography and CT with ^18^F-FDG

From the 1990s, PET has been extensively studied for use in the diagnosis and staging of cancer, especially lung cancer (Fischer *et al*., 2001). Initially as a single modality technique, it requires acquisition of both emission scan and transmission (for the purpose of attenuation correction), resulting in a PET scan from the skull base to upper thigh lasting approximately 45 min. In year 2000, the PET/CT scanner was introduced in the United States, and the first European scanners were installed in Copenhagen and Zürich in 2001. The introduction of the hybrid PET/CT scanner combining functional information from the PET scanner with anatomy and data for attenuation correction obtained by CT (Beyer *et al*., 2000) was a game changer for the PET technology. A whole-body PET/CT scan on modern scanners lasts approximately 20 min. Today, PET/CT is recommended in many oncological settings, especially in the diagnosis and staging of lung cancer. The PET technique is based on the tracer principle, and in this respect, [^18^F]-fluorodeoxyglucose (FDG) is by far the most commonly used PET tracer, exploiting the increased glucose uptake and metabolism in malignant cells (Pauwels *et al*., 2000). In the following text referring to PET and PET/CT, it is implicit that the tracer in question is FDG unless otherwise mentioned. The PET technology is inherently extremely sensitive, however, movement of the positron before annihilation and slight variations in the angle between the two photons limit the spatial resolution of current clinical systems to 4–5 mm (Sandler, 2003). This, combined with the unavoidable respiratory movements (PET acquisition takes approximately 3 min per bed position) have resulted in the rule of thumb that in tumours, smaller than 10 mm, the PET technique is considered to be less sensitive.

**Figure 1:**
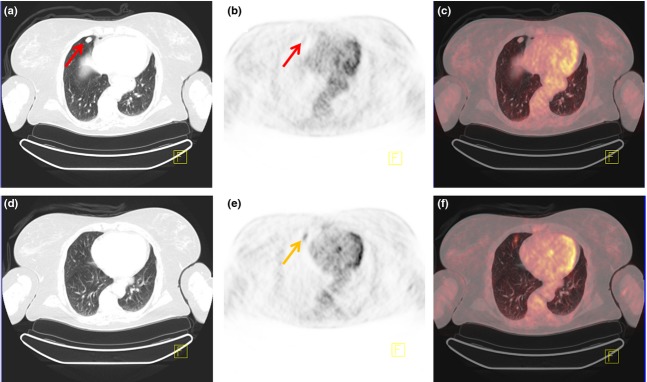
PET/CT scan of a 60-year-old female with a tumour anterior in the right lung, easily visible on CT (a). However, on PET/CT, FDG uptake was hardly visible in the area of the tumour (b,c). Taking a closer look at the slices above the tumour (d–f), moderately focal increased FDG uptake was visible here (SUV_max_ = 4). This uptake corresponds to the tumour, but as a result of respiratory movements during PET acquisition, there is a misalignment between CT and FDG-PET. The patient was diagnosed with a carcinoid tumour, which explains the relatively low FDG uptake.

Using PET in the diagnosis of lung cancer, a false-positive rate as high as 20–25% has been reported (Stroobants *et al*., 2003). This is mainly due to increased uptake of FDG in inflammatory cells (Fischer & Mortensen, 2006; Baxter *et al*., 2011). Also pulmonary embolism or iatrogenic microembolism can cause FDG uptake mimicking malignancy (but without correlate on CT) (Schreiter *et al*., 2011). Iatrogenic procedures might also induce false-positive results: of relevance in lung cancer is placement of chest tubes, percutaneous needle biopsy, mediastinoscopy, talc pleurodesis (may persist long after the procedure (Kwek *et al*., 2004)), radiation pneumonitis and esophagitis (Truong *et al*., 2005). Detailed knowledge of patient history and the use of integrated PET/CT (with side-by-side reading by nuclear medicine physician and radiologist) can help to distinguish malignant FDG uptake from uptake due to benign causes and improve specificity. False-negative results (Fig.1) are less common and mainly due to small size or well-differentiated malignancies, such as bronchiolo-alveolar adenocarcinomas and carcinoids (Marom *et al*., 2002; Higashi *et al*., 2003).

PET can be evaluated visually and/or semi-quantitatively by means of the standardized uptake value (SUV). SUV is the activity concentration in the lesion normalized for the injected dose and the weight or body surface area of the patient (Thie, 2004). SUV is highly dependent on a number of factors related to the patient (e.g. length of fast, period between injection of FDG and scan time), the type of scanner and reconstruction algorithm, making it unsuitable for uncritical comparison between different scanners, centres and time periods. A common SUV threshold suggesting malignancy cannot be recommended (Nguyen *et al*., 2011), and a PET report should always rely mainly on the visual analysis and only include information on the SUV to support the conclusion and if considered clinically relevant (Boellaard *et al*., 2010).

The CT part of the PET/CT scan is often performed as a whole-body low-dose scan without intravenous contrast (Pfannenberg *et al*., [Bibr b113][Bibr b114]). By doing this, it is possible to keep radiation dose from the CT scan at a minimum and avoid the use of IV contrast. It is, however, our experience and have also been demonstrated by recent studies that the diagnostic accuracy and clinical value of the PET/CT are markedly improved by applying a standard dose contrast-enhanced CT, also in the staging of patients with lung cancer, especially with regard to assessment of lymph nodes and for obtaining an accurate description of tumour extent (Pfannenberg *et al*., 2007b; Cronin *et al*., 2010).

### Dynamic contrast-enhanced CT

Dynamic contrast-enhanced CT is a functional imaging modality, which, in theory, can quantify the perfusion of tissues by calculating the delivery of contrast agent and therefore blood to these tissues (Miles *et al*., 1991, 1993, 1997). This is expected to be clinically useful, and accordingly, studies investigating the use of DCE-CT in oncology are increasingly reported in literature (Miles, 1999; Miles *et al*., 2000; Yi *et al*., 2004).

The fundamental principle of DCE-CT is based on the temporal changes in tissue density following intravenous administration of iodinated contrast media. By obtaining in quick succession a series of images of a particular tissue of interest, it is possible to record the temporal changes in the tissue attenuation occurring after intravenous injection of contrast. The quantification of perfusion recorded by CT is carried out using mathematical modelling techniques.

Dynamic contrast-enhanced CT has four fundamental requirements: (i) Intravenous administration of a contrast agent with a high flow rate, (ii) Repeated CT scans of the same volume of tissue. These scans must be obtained before, during and immediately after the intravenous administration of the contrast agent, to study the variation in tissue attenuation, (iii) Input of an arterial region of interest (ROI) to construct an arterial time–attenuation curve and (iv) Input of a tissue ROI to construct a tissue time–attenuation curve. The two curves are compared to obtain perfusion parameter measurements for the tissue interstitium of interest (Petralia *et al*., 2010a).

**Figure 2:**
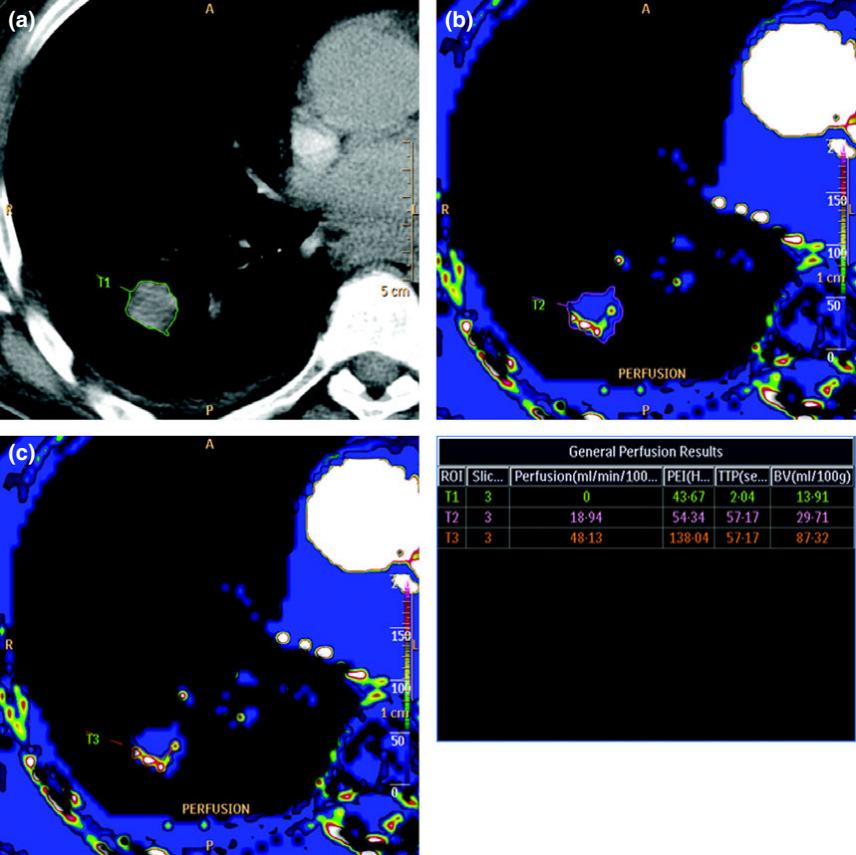
Examples of three different DCE-CT tumour ROIs using the quantitative method in a 69-year-old male with an adenocarcinoma in the right lung: (a) T1, a large ROI comprising the entire tumour. The ROI is drawn on a morphological image; (b) T2, a large ROI comprising the same entire tumour. This time, the ROI is drawn on a perfusion map; and (c) T3, a small ROI comprising only the maximally perfused parts of the tumour. The ROI is drawn on a perfusion map. These examples illustrate the significance of choosing the right ROI method and the importance of stating the chosen method in the report.

**Figure 3:**
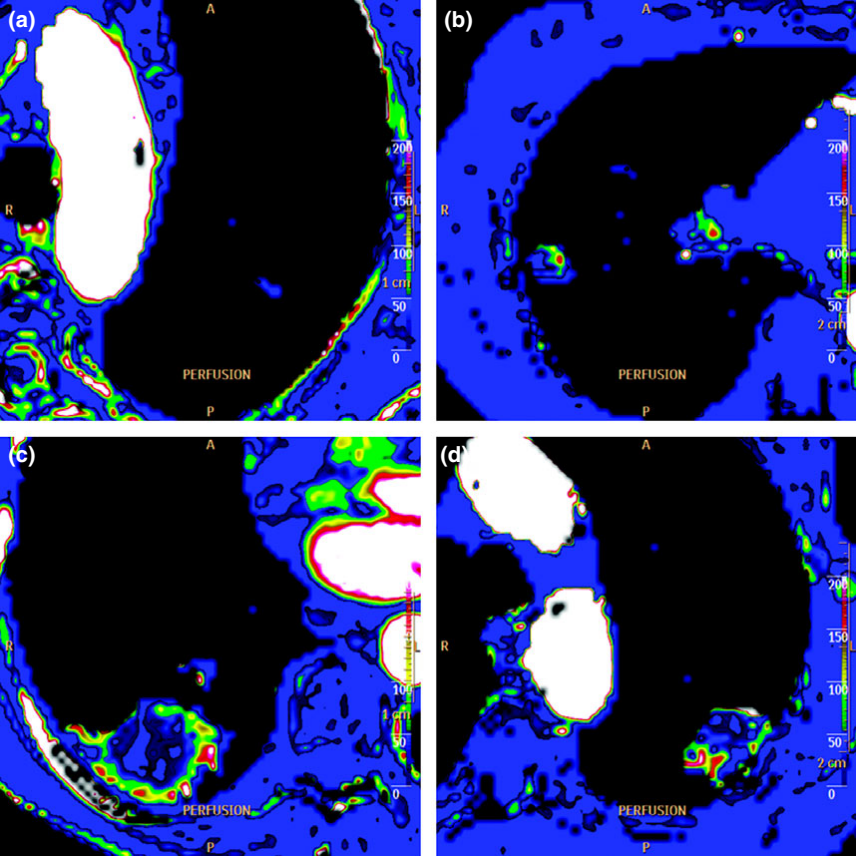
Four examples of enhancement patterns using a qualitative method: (a) No perfusion. The patient was a 79-year-old male with a squamous cell carcinoma. (b) Partial ring perfusion. The patient was a 66-year-old female with an adenocarcinoma. (c) Complete ring perfusion. The patient was a 67-year-old female with an adenocarcinoma. (d) Heterogeneous perfusion. The patient was a 78-year-old male with a squamous cell carcinoma.

Postprocessing of DCE-CT generates colour maps for both quantitative parameters, that is tumour blood flow and tumour blood volume, and semi-quantitative parameters, that is tumour peak enhancement intensity. In quantitative analysis, the operator places a ROI in the tumour, and dedicated perfusion software is then used to calculate numeric perfusion values for the ROI. This numeric value represents the mean of the numeric perfusion values for each voxel within the ROI, and as such, it provides an estimate of the total perfusion of the selected tumour volume (Fig.2). In qualitative analysis, the colour maps yield a visual impression of the blood flow and blood volume within the tissue being studied, allowing for quick identification of the areas with the highest or lowest blood flow and blood volume (Coche, 2012) (Fig.3).

The most important technical limitation of DCE-CT is respiratory motion, which can lead to image misregistration and errors in calculation of both quantitative and semi-quantitative parameters. Respiratory motion is a challenge for the actual parameters as well as for the reproducibility. This was evaluated in a study with 11 patients with lung tumour by Ng *et al*. (2011) using 16-detector row CT. The authors found that the quantitative parameters were significantly influenced by respiratory motion and the duration of data acquisition. Currently, the preferred method to minimize respiratory motion is to instruct patients to hold their breath or to use shallow breathing. However, motion correcting software is beginning to emerge both in descriptive as well as in comparative studies (Sauter *et al*., 2012a,b, 2013). In the future, the use of modern respiratory-gated, 256- or 320-detector row CT may improve misregistration through more extensive coverage, while reducing respiratory artefacts.

Another important limitation in DCE-CT concerns the radiation dose delivered to the patient and the use of potentially toxic intravenous iodinated contrast material. Fraioli *et al*. (2011a) measured the radiation dose to 21·7 ± 1·6 mSv using 64-detector row dual-source CT with tube voltage at 100 kV and tube current at 120 mAs. This is a substantial dose, similar to that of combined PET/CT.

### Diffusion-weighted magnetic resonance imaging

Recently, diffusion-weighted magnetic resonance imaging (DW-MRI) has become a widely used imaging modality, applied to evaluate tissue characterization in terms of its cellular density (tissue cellularity and the integrity of cellular membranes). DW-MRI is based on diffusion of water molecules in tissues. Water movement would be completely random in an unrestricted environment, a phenomenon known as Brownian motion (Koh & Padhani, 2006; Kwee *et al*., 2008). Within biological tissues, water molecules are distributed among intravascular, intracellular and extracellular spaces, and their motion is impeded by interaction with tissue compartment, cell membranes and intracellular organelles. In short, the more the viable cells, the higher the restriction of water diffusion.

The most common approach to render magnetic resonance imaging (MRI) sensitive to diffusion is by applying two strong symmetrical diffusion gradients on either side of the 180° refocusing pulse in the spin-echo sequence (Nasu *et al*., 2004). All water molecules of the imaged tissues will be affected by the first diffusion gradient, altering the phase shift of the protons in the water molecules; the second gradient reverses this phase shift, but only for those protons that have no spatial displacement during the acquisition time. Thus, if movement of the water molecule between application of the first and the second gradient pulses occurs, complete rephasing cannot happen leading to a loss of signal from this spatial location (Charles-Edwards & deSouza, 2006). On the other hand, in tissues with limited or almost no water diffusion, that is due to high cellularity as in malignant tumours, the MR signal will be retained. The sensitivity of the DWI sequence to water motion can be varied by changing the gradient amplitude, the duration of the applied gradient and the time interval between the diffusion gradients. The parameter proportional to these three factors is known as the b-value (Kwee *et al*., 2008; Qayyum, 2009). Diffusion-weighted imaging is performed with at least two b-values. By applying different b-values, quantitative analysis – known as the apparent diffusion coefficient (ADC) – is possible. Application of greater number of b-values improves the accuracy of ADC but increases the scanning time. As in all other existing modalities, false-positive and false-negative results do occur on DWI; with the most commonly occurring pitfalls being ‘T2 shine-through’ effect (delusions from slow flowing blood). Whereas DW-MRI measures cell density, FDG-PET/CT measures cell metabolism – thus, both modalities are prone to some of the same pitfalls hampering specificity, that is inflammation. DW-MRI has not yet been widely applied to diagnose, stage and therapy evaluation of patients with lung cancer due to artefacts from respiratory and cardiac motion. Low spatial resolution of DW-MRI images makes it difficult to evaluate a primary lesion and its relation to adjacent structures. However, use of standard MRI could help to delineate eventual spread into mediastinum, pleura and bone structures (Hochhegger *et al*., 2011; Biederer *et al*., 2012a,b). Scanning time for DWI is relatively short (the total imaging time could be around 228–265 s) (Hasegawa *et al*., 2008; Nomori *et al*., 2008; Nakayama *et al*., 2010) Concerning the procedure of DW imaging, using breath-hold technique can improve sensitivity (Biederer *et al*., 2012a,b), small nodules could, theoretically, be better visualized, and quantitative assessment of diffusion could be more accurately measured, but is still very dependent on the respiratory and heart rates, and data are currently scarce.

## Clinical results

### Lesion characterization

A solitary pulmonary nodule (SPN) is often the first depictable sign of lung cancer. However, an SPN is by no means equal to an early lung cancer. SPN is defined as a lesion smaller than 3 cm in diameter (larger than 3 cm is a mass) and completely surrounded by lung tissue (Wahidi *et al*., 2007). The probability of malignancy in an SPN varies significantly dependent on patient history (an incidental finding or a patient presenting with symptoms?), smoking history, age, radiological findings, etc. (Gould *et al*., 2007b). Assessing the pretest probability of malignancy in a given SPN facilitates clinical decision-making when selecting and interpreting the results of diagnostic imaging (e.g. PET) and invasive tests (Gould *et al*., 2007a).

#### Integrated positron emission tomography and CT with ^18^F-FDG

Early systematic reviews comparing the diagnostic value of PET compared to CT in discriminating malignant from benign pulmonary nodules or masses found PET to be highly sensitive (approximately 95%) but less specific (75–80%) (Fischer *et al*., 2001; Gould *et al*., 2001) and with positive and negative predictive values around 90%. The latter depends on the prevalence or pretest probability of cancer. By applying the likelihood ratio, it can be seen that a negative PET scan (LR = 0·05) results in a large gain of knowledge from pre- to post-test probability of cancer as compared to a negative CT (LR = 0·2) (Fischer *et al*., 2001). In short, in the setting of pulmonary nodules, PET is better to exclude than confirm malignancy. More recent studies comparing CT and PET/CT are scarce but can reproduce a significant difference between CT and PET/CT, in favour of the latter (Yi *et al*., 2006; Kim *et al*., 2007; Jeong *et al*., 2008; Kagna *et al*., 2009). This difference is seen both with regard to sensitivity and specificity, but especially the difference in specificity decreases (Harders *et al*., 2012), when comparing PET/CT with standard contrast-enhanced CT. These studies are, as well as the earlier studies on the diagnosis of lung nodules, hampered by a relatively high prevalence of cancer (40–70%) (Fischer & Mortensen, 2012). Thus, the high diagnostic accuracy of PET/CT for diagnosing SPN found in these studies may not be valid in the present population where an increasing number of patients are referred with an incidental finding of a solitary pulmonary nodule, for exmaple, after performing a cardiac CT due to angina or chest X-ray during a health examination or as a result of participation in a lung cancer screening trial. The ability of PET to rule out a malignant diagnosis can potentially reduce the number of invasive procedures resulting from, for example, cardiac CT and screening trials (Pastorino *et al*., 2003). However, in a screening population, an increased frequency of small and relatively low metabolic tumours can be expected, hampering the sensitivity and specificity of the PET technique (Lindell *et al*., 2005; Bar-Shalom *et al*., 2008). This problem can be addressed by combining the information of tumour growth rate (e.g. after 3 months) with FDG uptake (Bastarrika *et al*., 2005; Ashraf *et al*., 2011), making PET/CT a valuable second-step test. Large and well-designed trials addressing this issue are still scarce.

#### Dynamic contrast-enhanced CT

Early reports (Zhang & Kono, 1997; Yi *et al*., 2004) described how blood flow and peak enhancement intensity were higher for malignant and inflammatory tumours than for benign ones. A subsequent meta-analysis found the diagnostic accuracy of DCE-CT, DCE-MRI, FDG-PET and 99mTc depreotide single-photon emission CT (SPECT) for the evaluation of solitary pulmonary nodules to be comparable, with only negligible differences in performance between the tests (Cronin *et al*., 2008).

Since these reports, DCE-CT methods have been sophisticated, and quantitative and semi-quantitative methods of analysis have been further developed to better characterize the nature of lung nodules and tumours (Chae *et al*., 2008, 2010; Li *et al*., 2010; Ohno *et al*., [Bibr b101][Bibr b102]). Thus, in a recent report, Sitartchouk *et al*. (2008) demonstrated higher blood flow, blood volume and extraction fraction values in malignant lung nodules compared with benign ones, and in another report, Ohno *et al*. ([Bibr b101][Bibr b102]) used 256- and 320-detector row CT to acquire dynamic data within a 16-cm area every 2 s without helical scanning. In 50 patients with 76 lung nodules, the authors showed that first-pass area-detector DCE-CT had the potential to be more specific and accurate than FDG-PET/CT for differentiating between malignant and benign lung nodules.

#### Diffusion-weighted magnetic resonance imaging

**Figure 4:**
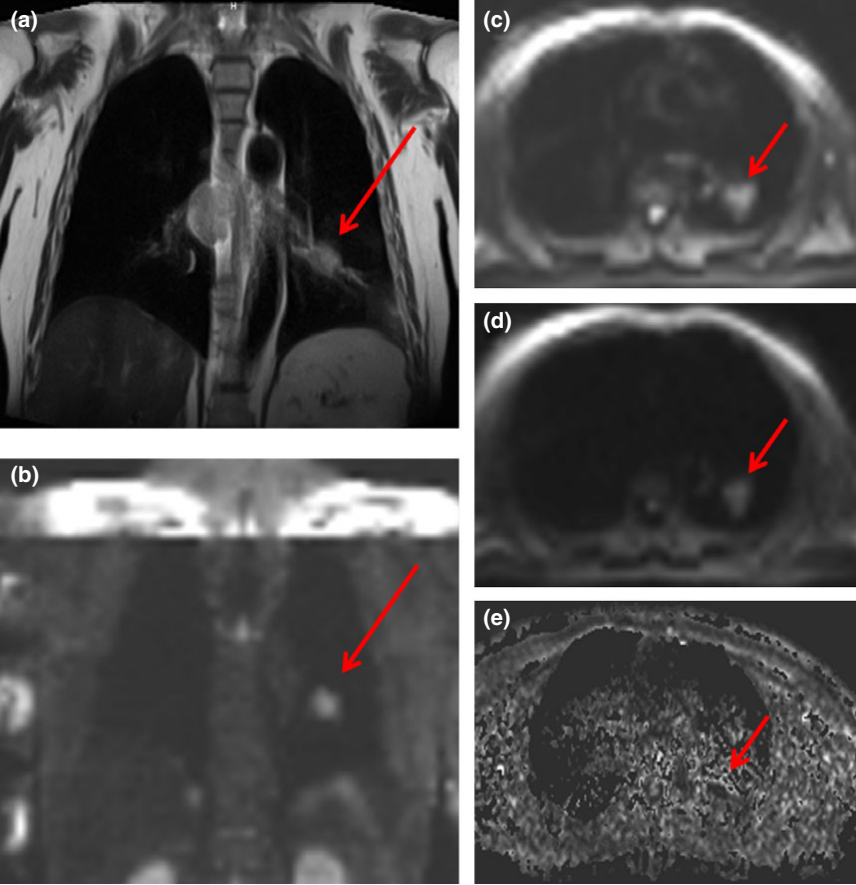
MR scans of a 54-year-old patient with adenocarcinoma in the left lung. During initial examination on coronal T2-weighted images, (a) primary tumour (red arrow) is seen as being isointense to soft tissue. Correspondent coronal DW image displays tumour as a hyperintense zone (b). On the transaxial images, the SI (signal intensity) of the primary tumour (on *b*-value of 50 and 1000 s mm^−2^) is seen to stay relatively high and only decrease slightly together with lesion diameter if *b*-value is elevated (c and d). On ADC (e) map, the primary lesion shows low SI, corresponding to the malignant nature of the tumour.

Only few studies have assessed the value of DW-MRI for solitary pulmonary nodules: Ohba *et al*. (2009) found that DWI clearly identified the malignant nodules in a fashion similar to PET imaging. Similarly, Mori *et al*. (2008) compared the performance of PET and DW-MRI using cut-off values for respectively SUV and ADC. They found the two modalities to be equally sensitive (0·72 and 0·70, respectively), whereas DW-MRI was significantly more specific (0·97 and 0·79, respectively). At the moment, there is no standard ADC threshold for differentiation benign from malignant lesion. On high *b*-values (1000 s mm^−2^), tissues with restricted diffusion appear bright on DW images and dark on the ADC map; if several high *b*-values are applied, malignant lesions will show increased signal intensity (SI) with increasing *b*-values (Fig.4). By some authors this correlation between increasing *b*-values and SI is considered to be a useful marker of malignancy, that is, when differentiating metastases from haemangioma in the liver (Parikh *et al*., 2008; Inan *et al*., 2010). In a recent study, SI was significantly different between malignant and benign lung lesions, whereas there was no significant difference in ADC values between malignant and benign lesions (Gumustas *et al*., 2012).

#### Recommendations

In accordance with recent recommendations, PET/CT should be used for evaluation of patients with pulmonary nodules with low to moderate risk of malignancy (solid nodules 8–30 mm in size and indeterminate on CT) (Gould *et al*., 2013). Recent data indicate that DCE-CT could be a possible alternative to PET/CT for this indication, but more studies are needed to confirm this. Data on DW-MRI are promising but scarce, and the field lacks standardization.

### Lung cancer staging

Treatment options for patients with lung cancer are highly dependent on the stage of the disease, making accurate and fast staging pivotal. Non-small-cell lung cancer is staged according to the TNM system as initially suggested by Mountain (Mountain, 1986, 1997) and recently revised by the International Association of Lung Cancer (Detterbeck *et al*., 2009). Staging is used to predict survival and to guide the patient towards the most appropriate treatment regimen or clinical trial. The most significant division is between those patients who are candidates for surgery and those who may benefit from chemotherapy, radiation therapy or both. Only patients with localized disease (TNM stage I-IIb evt. IIIA) will be candidates for primary curative surgery. For most patients with advanced disease (stage IV), palliative treatment with chemotherapy will be the only option. Thus, to allocate the patient to the correct treatment, accurate description of (i) distant metastases and (ii) mediastinal spread (N) is mandatory, whereas the T-stage will substantially influence the treatment choice only in the case of tumour invasion making resection impossible.

#### Integrated positron emission tomography and CT with ^18^F-FDG

**Figure 5:**
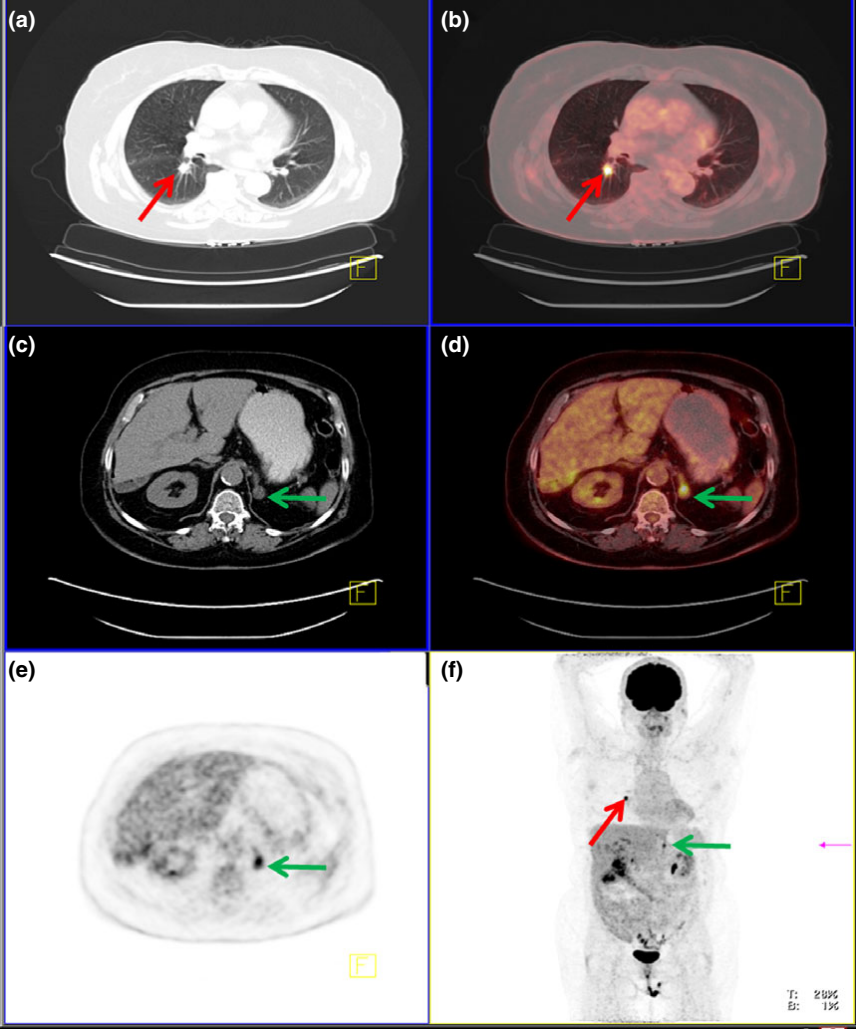
PET/CT scan of a 66-year-old female with a central tumour in the right lung (red arrow), easily visible on CT (a). Tumour was highly FDG-avid indicative of malignancy (SUV_max_ = 16) (b). An enlarged left adrenal gland was visible on CT (green arrow) (c) and similar to the primary tumour highly FDG-avid, corresponding to a metastasis (SUV_max_ = 9) (d,e). Both findings can be seen on the multi-intensity projection (MIP, f) and was confirmed to be adenocarcinoma.

Single modality PET is insufficient for an accurate description of T-stage, whereas combined PET/CT is significantly more accurate than both PET (Lardinois *et al*., 2003; Cerfolio *et al*., 2004; Halpern *et al*., 2005) and standard CT (diagnostic quality with intravenous contrast) for T-staging (Antoch *et al*., 2003; de Wever *et al*., 2007). *Mediastinal staging* is, in patients without distant metastases, the most significant factor for treatment planning as mediastinal spread (N2–N3 disease) excludes the patient from primary surgery. Initial reports on PET reported very high accuracy with regard to N-staging and significantly higher than the accuracy of CT (Fischer *et al*., 2001; Reed *et al*., 2003). In more recent studies, this difference seems to narrow down, but still a staging strategy including PET/CT appears more sensitive with regard to mediastinal disease (de Wever *et al*., 2007; Fischer *et al*., 2011). It has been suggested that mediastinoscopy or other invasive staging can be omitted in cases where mediastinum is PET negative (Detterbeck *et al*., 2007; de Leyn *et al*., 2007). But by doing this, 16% of the patients have occult N2 disease (Al-Sarraf *et al*., 2008). To avoid this, patients with central tumours, enlarged lymph nodes on CT and/or N1 disease on PET/CT should perform a confirmatory invasive examination (Fischer *et al*., 2011). *Distant metastases* (M1 disease) in otherwise operable patients are reported in approximately 5–15% of patients performing PET or PET/CT (Fig.5) (van Tinteren *et al*., 2002; Lardinois *et al*., 2003; Reed *et al*., 2003; Fischer *et al*., 2009). Assessing the overall diagnostic accuracy of PET/CT, PET and CT with regard to M-stage is hampered by limited relevant literature as well as protocol variations. A high diagnostic value of PET/CT for diagnosing bone metastases is well documented, and PET/CT is found to be more sensitive than bone scan and CT (Fischer *et al*., 2007; Song *et al*., 2009). Similarly PET, and recently PET/CT, is effective in discriminating between malignant and benign adrenal masses (Metser *et al*., 2006; Ozcan Kara *et al*., 2011). Isolated PET-positive lesions should however be confirmed to avoid deeming a patient inoperable on a false-positive basis. Due to the high background signal caused by physiological cerebral FDG uptake, PET performs poorly in the detection of brain metastases, especially compared to cerebral MRI. Kruger *et al*. (2011) found a sensitivity for brain metastases of 27% in 104 lung cancer patients with neurological symptoms. PET/CT can detect brain metastases, but a negative PET/CT scan does not exclude brain metastases, especially not in case of neurological symptoms.

The clinical value of PET/CT for pre-operative staging has been demonstrated by two randomized studies. Both studies found that pre-operative staging with PET/CT significantly reduces the frequency of futile thoracotomies without affecting overall survival (Fischer *et al*., 2009; Maziak *et al*., 2009). The question of whether or not the use of PET/CT improves survival, apart from improved stage-specific survival as a result of stage migration, has recently been discussed (Dinan *et al*., 2012; Gregory *et al*., 2012; Hofman *et al*., 2013). As mentioned above, one of the important effects of applying PET and PET/CT to lung cancer staging is the detection of unrecognized metastases and upstaging of patients. This results in more patients receiving palliative treatment instead of potentially curative therapy, that is surgery. If PET inappropriately upstages patients, overall survival should decrease. Thus, as stated by Hofman *et al*., finding that all-cause survival does not change significantly with the increasing use of PET supports a conclusion that PET may reduce morbidity associated with futile therapies without negatively affecting overall patient outcomes.

#### Dynamic contrast-enhanced CT

Standard contrast-enhanced CT is the most widely available and commonly used non-invasive modality for the evaluation of the mediastinum in lung cancer. Forty-three studies evaluating the accuracy of CT scanning for staging the mediastinum were analysed in the American College of Chest Physicians (ACCP) guidelines from 2013 (Silvestri *et al*., 2013). The pooled sensitivity and specificity of CT scanning for identifying mediastinal lymph node metastasis were 55% and 81%, respectively. Although the combined estimates should be interpreted with caution as the studies were statistically heterogeneous, these findings closely mirrored previous analyses addressing the accuracy of CT scanning for staging the mediastinum in NSCLC by Gould *et al*. (2003) and by Dwamena *et al*. (1999). While it remains the best overall anatomical study available for the thorax, CT is clearly an imperfect means of staging the mediastinum. Thus, CT both overstages as well as understages the mediastinal nodes. Nonetheless, CT continues to play an important and necessary role in the evaluation of these patients.

Dynamic contrast-enhanced CT has been studied as a means of identifying patients with lung cancer at risk of malignant nodal infiltration. However, conflicting results have been reported so far. In a retrospective study of 130 patients with NSCLC who underwent pre-operative DCE-CT followed by surgical resection, Tateishi *et al*. (2002) reported that tumour peak enhancement intensity was significantly higher in patients with lymph node involvement compared with patients without nodal involvement. Likewise, preliminary studies by Li *et al*. ([Bibr b74][Bibr b75]) involving patients with surgically resected lung cancer showed a trend towards higher tumour blood flow and peak enhancement intensity when nodal infiltration was present. Unfortunately, however, these results were not statistically significant when a larger cohort of patients were analysed (Li *et al*., 2008b). At present, DCE-CT has no role in the detection of extra thoracic metastases.

#### Diffusion-weighted magnetic resonance imaging

In recent years, DW-MRI has been brought forward as a potential tool in oncology to differentiate benign from malignant lymph nodes (Qayyum, 2009; Malayeri *et al*., 2011; Bonekamp *et al*., 2012). Several studies have demonstrated that this technique could be used to distinguish metastatic from non-metastatic lymph nodes in patients with NSCLC (Hasegawa *et al*., 2008; Nomori *et al*., 2008; Nakayama *et al*., 2010). On high b-values, malignant nodes could have slightly different SI (signal intensity) compared to the primary lesion. In general, under quantitative assessment of DW-MR images of lymph nodes, the ADC is found to be significantly lower for metastatic lymph nodes; however, no broadly accepted ADC threshold exists (Ohno *et al*., 2011a,b). In a recent meta-analysis comparing DW-MRI and FDG-PET/CT, the authors found equal sensitivity (PET/CT 0·75 and DW-MRI 0·72), but a higher specificity for DW-MRI compared to PET/CT (0·95 and 0·89, respectively) (Wu *et al*., 2012). Two recent clinical trials could not reproduce any difference in the diagnostic value of PET/CT and DW-MRI for staging of NSCLC (Pauls *et al*., 2012; Sommer *et al*., 2012), thus, for the moment, DW-MRI might be considered as a supplement or maybe even a substitution technique for FDG-PET/CT in staging of patients with lung cancer.

#### Recommendations

For staging of patients with lung cancer imaging, assessing potential locoregional as well as extrathoracal spread is needed. Staging by means of PET/CT (supplemented with invasive examination of eventual mediastinal spread) is currently the state of art (Silvestri *et al*., 2013). Whether invasive mediastinal staging can be omitted in patients with small tumours and negative mediastinum on PET and CT is likely but still controversial. Data on whole-body DW-MRI for lung cancer staging are emerging and promising but needs confirmation in larger studies. As for now, there is no role for DCE-CT for staging of lung cancer.

### Treatment monitoring

The definition of tumour response using the WHO (Miller *et al*., 1981) and RECIST (Eisenhauer *et al*., 2009) criteria is based upon an experiment performed 30 years ago determining the accuracy with which sixteen experienced oncologists could measure tumour size by palpation (Weber, 2005). Structural imaging techniques such as chest radiographs and CT provide excellent anatomical details and are essential tools in the care of patients with lung cancer. However, whether structural imaging is the most valid measure of tumour response is uncertain (Vansteenkiste *et al*., 2004), and data suggest that measurement of lung tumour size on CT scans is often inconsistent (Erasmus *et al*., 2003). Furthermore, structural changes after surgery and/or radiotherapy can be difficult to discern from local tumour relapse and new generations of targeted drugs does not necessarily result in significant structural decreases, despite clinical effect.

#### Integrated positron emission tomography and CT with ^18^F-FDG

Data on several solid tumours, including non-small-cell lung cancer, indicate a possible advantage of response evaluation by FDG-PET during and after chemotherapy (Juweid & Cheson, 2006; Herrmann *et al*., 2011). As FDG preferentially accumulates in viable tumour cells and not in fibrotic or necrotic tissue (Higashi *et al*., 1993), a change in FDG uptake on PET could be a better way to monitor response and perhaps even to assess response before structural changes occur. Current evidence indicates that FDG-PET response has a variable correlation with CT response, but probably is more accurate than CT response (Maziak *et al*., 2009). The first clinical study on PET for therapy evaluation in patients with NSCLC was published in 2003. Weber *et al*. (2003) examined 57 patients before and after one cycle of chemotherapy, demonstrating that changes in tumour FDG retention was closely correlated to prognosis. The principle of using PET for (early) therapy evaluation has since been tested in numerous studies and settings, including conventional cytotoxic chemotherapy, radiotherapy and molecular-targeted therapy, that is EGFR tyrosine kinase inhibitors (Hoekstra *et al*., 2002; Mac Manus *et al*., 2005; van Baardwijk *et al*., 2007; Kong *et al*., 2007; Maziak *et al*., 2009; Benz *et al*., 2011). All studies conclude that PET is potentially useful for therapy planning and evaluation; however, larger controlled trials assessing the clinical effect is still lacking as is necessary standardization of SUV measurements (Weber & Figlin, 2007).

#### Dynamic contrast-enhanced CT

Several studies have suggested that DCE-CT may be potentially useful in the assessment of patients undergoing chemotherapy, radiation therapy and ablation therapy (Kiessling *et al*., 2004; Wang *et al*., 2009; Bellomi *et al*., 2010; Hegenscheid *et al*., 2010; Lind *et al*., 2010; Petralia *et al*., 2010a,b; Tacelli *et al*., 2010; Fraioli *et al*., 2011a,b). Thus, case studies have revealed changes in quantitative and semi-quantitative parameters in patients with NSCLC who were treated with ‘non-vascular targeting’ agents. In preliminary studies, Wang *et al*. (2009) found a significant decrease in tumour blood flow and blood volume in a patient following two cycles of chemo- and radiotherapy, while Kiessling *et al*. (2004) described a reduction in tumour blood flow in a patient after two cycles of chemotherapy. The effects of combined chemotherapy and anti-angiogenic agents have also been investigated (Lind *et al*., 2010; Fraioli *et al*., 2011a). Angiogenesis and epidermal growth factor receptor inhibitors were evaluated in a study including 23 patients who received dual-source CT at baseline and 3 and 6 weeks after treatment. In their report, Lind *et al*. described how mean tumour blood flow decreased significantly from 39·2 ml per 100 g min^−1^ at baseline to 15·1 ml per 100 g min^−1^ at week 3–9·4 ml per 100 g min^−1^ at week 6. Tumour blood flow was lower in RECIST responders versus non-responders at week 3 and 6, respectively (Lind *et al*., 2010). In another study, Fraioli *et al*. (2011a) assessed 45 patients with non-resectable NSCLC > 20 mm. Subjects underwent DCE-CT at baseline and 40 days after treatment with chemotherapy and anti-angiogenic agents. The authors showed how treatment-induced changes in perfusion could be identified using DCE-CT. They also found that tumour blood flow, blood volume and permeability values were lower in responders compared with non-responders. Of particular interest, they observed discrepancies between quantitative and semi-quantitative assessments, and RECIST criteria evaluations. The authors emphasized the fact that macroscopic changes in tumour size did not necessarily reflect the biological changes induced by therapy. It is thus possible that DCE-CT performed shortly after initiating therapy may be useful for therapy planning, as it may provide a better evaluation of physiological changes than the conventional size assessment obtained using RECIST criteria.

#### Diffusion-weighted magnetic resonance imaging

Apparent diffusion coefficient seems to be a promising tool for an early tumour response assessment. Derived from DW-MRI, ADC has been shown to be a useful biological marker for early detection and prediction of tumour response to chemotherapy and chemoradiotherapy in different malignant tumours, including head and neck, brain tumours, hepatic metastasis and gynaecological tumours (Harry *et al*., 2008; Kim *et al*., 2009). A study dedicated to early response detection to chemotherapy by DCE- and DW-MRI showed that an increase in ADC after one course of chemotherapy (increase by more than 25% from initial ADC value) correlated with longer progression-free survival (Biederer *et al*., 2012a,b). Results of this study suggest that early response to therapy could be predicted by means of ADC change, despite the absence of any obvious morphological changes (e.g. shrinkage of tumour size or anatomical changes in tumour structure); this could, probably, be explained by early alterations of tumour cellular density under anticancer drugs therapy, which increases volume of tumour extracellular spaces by means of cells necrosis and apoptosis (Koh & Padhani, 2006; Hochhegger *et al*., 2011). It has been hypothesized that tumours with low baseline pretreatment ADC values respond better to chemo- or radiotherapy than tumours with high ADC values. Tumours with high pretreatment ADC values are more likely to be necrotic, leading to decreased sensitivity to treatment. This is confirmed in a recent study by Ohno *et al*. (2012) including 64 patients with NSCLC, in which the ability of DW-MRI (ADC values) and FDG-PET/CT (SUVmax), respectively, to predict response to chemo radiotherapy was assessed. The authors found that both ADC and SUVmax could significantly differentiate between responders and non-responders. However, performing a ROC analysis, the area under the curve (AUC) was significantly larger when using ADC compared to SUV, suggesting that DW-MRI might have better potential for prediction of tumour response than FDG-PET/CT.

#### Recommendations

Data on functional imaging in therapy evaluation of patients with lung cancer are characterized by many smaller and promising studies, but randomized studies or studies assessing the potential impact on survival of functional imaging for this indication are lacking. Structural assessment by RECIST still is the gold standard, but with targeted therapy, targeted or tailored evaluation should follow, for example using DCE-CT for the evaluation of treatment with anti-angiogenic drugs, DW-MRI for drugs causing apoptosis and PET/CT for drugs targeting metabolism and proliferation. Common for all three modalities is an urgent need for standardization.

## Discussion

In this paper, we have described three functional imaging modalities for use or potential use in the care of patients with lung cancer (Table1). The amount of evidence on the value of PET and PET/CT for diagnosing and staging lung cancer is huge, and PET now has its place in several recommendations and guidelines for this indication. In many of the studies cited in this paper, PET/CT is used as a reference standard for comparison with the newer modalities such as DCE-CT and DW-MRI. It is intriguing to compare these functional modalities, but it should be stressed that while all three modalities are indeed functional they measure different tissue characteristics, that is metabolism, perfusion and cellularity. By focusing only on their respective diagnostic value, we might miss important information from the combined assessment of different tissue characteristic (Kim *et al*., 2012).

Dynamic contrast-enhanced CT is a promising imaging modality in lung cancer. A number of smaller studies have successfully identified patients with lung cancer, patients with nodal involvement and patients who might benefit from specific treatment regimens. Only few reports have compared F-18-FDG-PET/CT with DCE-CT. A complex relationship between tumour glucose metabolism and tumour blood flow has been hypothesized (Miles & Williams, 2008). Thus, in a study of standardized uptake value (SUV) measured using F-18-FDG-PET and standardized perfusion value (SPV) measured using DCE-CT, Miles *et al*. reported a positive correlation between the ratio of SUV to SPV in lung cancer with higher values found in larger tumours. The authors also reported a significant correlation between SUV and SPV for tumours smaller than 4·5 cm^2^ (Miles *et al*., 2006).

Dynamic contrast-enhanced CT is challenged by technical difficulties, lack of reproducibility and a rather high radiation dose. Therefore, at present, the modality cannot be recommended for standard clinical use in suspected or known lung cancer. Future research and development should preferably include automatic segmentation of tumours with an acceptable reproducibility and tools to reduce motion artefacts. Until then, DCE-CT remains experimental and should only be used in research and in special situations.

Diffusion-weighted magnetic resonance imaging has already shown its usefulness in differentiation, response assessment and recurrence disease identification in primarily brain, head and neck, abdominal and pelvic tumours. Diagnostic value of lung MR imaging is still interrogative, but lack of radiation and no need for contrast material to be used in DW-MRI makes this technique very attractive, especially in cases where repetitive examinations should be performed. DWI could also be applied for patients with renal dysfunction. Early changes of the ADC values before evident morphological tumour tissue alterations could help to conduct chemo- and chemoradiotherapy in a more efficient way. Low spatial resolution is still considered to be a problem and probably could be overcome with the help of fusion T2 and DW images; in these terms, new techniques to subdue challenging MR respiratory and cardiac motion artefacts should be found.

More and more studies comparing DW-MRI and FDG-PET/CT are emerging. In a recent study comparing the performance of DW-MRI and FDG-PET/CT in mediastinal staging, the specificity of both DW-MRI and FDG-PET was hampered by the occurrence of inflammatory lymph nodes. However, only FDG-PET was false positive in the presence of anthracosilicosis, whereas DW-MRI was false positive in the presence of lymphatic oedemas and coagulation necrosis (Ohno *et al*., 2011a,b). Exciting data derived from a rat glioma model examining the treatment-associated inflammatory response by DW-MRI and FDG-PET suggest less profound effect of chemotherapy-associated immunological response on tumour diffusion compared to tumour FDG uptake after therapy (Galban *et al*., 2010). PET/MR would be the method of choice for a more thorough assessment of the differences and correlation between FDG-PET and DW-MRI, and implications for diagnosis and response evaluation.

## Conclusion and perspectives

PET/CT still is a rational first choice for diagnosing and staging patients with lung cancer, however, data are emerging that DCE-CT could be a reasonable alternative or supplement for assessment of pulmonary nodules and perhaps mediastinal staging. Data are emerging that staging by DW-MRI could be a valuable alternative to PET/CT. For therapy evaluation, data are less mature for all three modalities, but for future clinical trials in this field, we should try to consider the three modalities not as competitors, but as complements evaluating different hallmarks of cancer biology. Especially within the field of therapy prediction and evaluation, an intelligent combination of different functional imaging modalities could prove very valuable (Reed *et al*., 2003), but also the addition of an extra functional parameter could increase diagnostic accuracy in many settings (Kim *et al*., 2012). With a more sophisticated use of combined PET/CT scanners and the emergence of PET/MR, this is not a pipe dream but can be done now.

## Conflict of interests

The authors have no conflict of interest.
